# Benchmarking of microbiome detection tools on RNA-seq synthetic databases according to diverse conditions

**DOI:** 10.1093/bioadv/vbad014

**Published:** 2023-02-22

**Authors:** Francisco Jurado-Rueda, Lola Alonso-Guirado, Tomin E Perea-Chamblee, Oliver T Elliott, Ioan Filip, Raúl Rabadán, Núria Malats

**Affiliations:** Genetic & Molecular Epidemiology Group, Spanish National Cancer Research Centre and CIBERONC, Madrid 28029, Spain; Genetic & Molecular Epidemiology Group, Spanish National Cancer Research Centre and CIBERONC, Madrid 28029, Spain; Program for Mathematical Genomics and Department of Systems Biology, Columbia University, New York, NY 10027, USA; Program for Mathematical Genomics and Department of Systems Biology, Columbia University, New York, NY 10027, USA; Program for Mathematical Genomics and Department of Systems Biology, Columbia University, New York, NY 10027, USA; Program for Mathematical Genomics and Department of Systems Biology, Columbia University, New York, NY 10027, USA; Genetic & Molecular Epidemiology Group, Spanish National Cancer Research Centre and CIBERONC, Madrid 28029, Spain

## Abstract

**Motivation:**

Here, we performed a benchmarking analysis of five tools for microbe sequence detection using transcriptomics data (Kraken2, MetaPhlAn2, PathSeq, DRAC and Pandora). We built a synthetic database mimicking real-world structure with tuned conditions accounting for microbe species prevalence, base calling quality and sequence length. Sensitivity and positive predictive value (PPV) parameters, as well as computational requirements, were used for tool ranking.

**Results:**

GATK PathSeq showed the highest sensitivity on average and across all scenarios considered. However, the main drawback of this tool was its slowness. Kraken2 was the fastest tool and displayed the second-best sensitivity, though with large variance depending on the species to be classified. There was no significant difference for the other three algorithms sensitivity. The sensitivity of MetaPhlAn2 and Pandora was affected by sequence number and DRAC by sequence quality and length. Results from this study support the use of Kraken2 for routine microbiome profiling based on its competitive sensitivity and runtime performance. Nonetheless, we strongly endorse to complement it by combining with MetaPhlAn2 for thorough taxonomic analyses.

**Availability and implementation:**

https://github.com/fjuradorueda/MIME/ and https://github.com/lola4/DRAC/.

**Supplementary information:**

Supplementary data are available at *Bioinformatics Advances* online.

## 1 Introduction

Microbes live and share with us a long history of evolution. Most of them are key allies for certain molecule transformations and further assimilation, e.g. plants origin complex polysaccharides (Flint *et al.*, 2012). Others are vital maintainers of tissue homeostasis through certain metabolite generation such as bacterial production of butyrate and its anti-inflammatory role (Chen *et al.*, 2019). However, other microbes are potentially lethal pathogens with a number of causal relationships having been identified, e.g. *Helicobacter pylori* associates with gastric cancer and Human Papilloma Virus (HPV) with cervical cancer (Yuan *et al.*, 2020). Therefore, microbe detection is a cornerstone in clinical research either for taking advantage of their benefits or to minimize their opportunistic harmful role.

The traditional approaches for microbe detection process relied on cultured-based methods. These strategies had serious limitations regarding time and scaling optimization due to manual procedures. Nowadays, thanks to high throughput technologies, such as culturomics and next generation sequencing (NGS), most of the previous limitations are overcome. Nonetheless, new challenges appeared in the microbe identification task derived from the in silico set up these new technologies require. All the available state-of-the-art bioinformatics tools rely on specific algorithms to correctly classify microbial sequences (i.e. either metatranscriptomics, metagenomics or 16S rRNA sequencing). Despite their uniqueness, bioinformatics tools can be roughly categorized into binners and classifiers, depending on their strategies. While classifiers take the input as a whole and work with sequences collectively resulting in a summary of the presence and relative abundance of taxa, binners focus on one read at a time and allocate it individually to the microbe sequence offering quantitative results per sequence. Differences between their outputs formats can be observed that a tool’s user notices (Sczyrba *et al.*, 2017). The challenge of identifying microbes is a constantly evolving hot-topic, applicable to ecology profiling, individual medicine diagnosis, etc. Therefore, a formal comparison of the available bioinformatics tools needs to be performed to identify the best fitting algorithm and keep track of new promising strategies. While several benchmarking studies have been previously conducted, some of them were focused on presenting new metagenomics classifier tools, devising *ad hoc* datasets to highlight their attributes (Andrusch *et al.*, 2018; Gihawi *et al.*, 2019; Saha *et al.*, 2019). The rest of the benchmarking papers focused their effort on taking on board as many tools as possible (Ye *et al.*, 2019), comparing hundreds of newly sequenced genomes (Sczyrba *et al.*, 2017), or comparing biological vs. simulated datasets (McIntyre *et al.*, 2017). Here, we present the first benchmarking that focuses its main effort to assess sequence conditions’ impact on the tool’s performance. In this study, we addressed these limitations and compared the performance of three publicly available tools for microbiome detection (Kraken2, MetaPhlAn2 and GATK PathSeq) and two in-house tools (DRAC and Pandora). Importantly, we constructed a valuable synthetic database with controlled and changing conditions accounting for bacterial prevalence, read base calling quality and sequence length, and we evaluated how these conditions affected each tool’s performance. In summary, we observed that Kraken2 balances well between low runtime requirements and competitive sensitivity making it a suitable tool for regular microbial profiling. For in-depth studies, we recommend the use Kraken2 jointly with MetaPhlAn2 due to their species-specific complementation.

## 2 Materials and methods

### 2.1 The algorithms

The three publicly available in silico tools that we compared (Kraken2, MetaPhlAn2 and GATK PathSeq) were selected due to their recurrent usage in microbiome sequences recognition. Each of the selected tools relies on a state-of-the-art algorithm and, in combination, the three of them cover the main strategies for the taxonomic sequence classification and taxonomic label assignation (Table 1). In addition, these tools recurrently appear in most of the previous benchmarking studies. Besides, many of the new tools released to these purposes prove their performance by comparing them with the three selected algorithms (Gihawi *et al.*, 2019). Briefly, Kraken2 (cited 797 times in PubMed) is a binner tool based on an exact ‘k-mer’ alignment. Kraken’s database can be custom-generated, tailored to recognize specific taxa. Kraken2 uses a compact hash table with each k-mer being associated with a taxon. It splits each query sequence into n pieces called k-mers (31-bases long by default). It works with one k-mer at a time, assigning it to a particular taxon. The final label of a given sequence is computed out of the weighted average of all its k-mers. In case of a tie, it would be labeled to the lowest common ancestor (LCA) (Wood *et al.*, 2019). MetaPhlAn2 (cited 939 times in PubMed) is a classifier tool based on marker genes. Its database is a reduced collection of characteristic genes preselected from coding sequences that unequivocally identify specific microbes. Hence, no previous read filtering is required. The aligner matches the raw sequences to the catalog of marker genes with a threshold of 75% of identity (Segata *et al.*, 2012). GATK PathSeq (GATK) is also a binner tool (cited 190 times in PubMed). It is based on three consecutive filters. It discards low quality, low complexity, duplicated and human-related reads. For this purpose, it uses several filters such as DUST for low-complexity sequences and Bloom filters for addressing false positive probability. This step removes most of the human reads; while even the reads with a single human k-mer are discarded by default, this criterion can be changed with the parameter host k-mer count threshold (Walker *et al.*, 2018). The second filter is carried out by BWA-MEM aligner which uses human genome as a reference to get rid of any remaining read that might have passed the previous filter. The last filter applies BWA-MEM again and queries different microbial genomes in order to classify taxonomically the non-human remaining reads (Walker *et al.*, 2018). Two recently developed tools (DRAC and Pandora) were included in the benchmarking study as they overcome some of the limitations from the previous ones. DRAC (discarded reads alignment and coverage) was developed by a co-author of this article (LA, Supplementary Material). It is a binner tool that takes a BAM file as input and dismisses the human-mapped sequences filtering by the bitwise flag. Then, it maps the rest of the sequences against a reference database (bacterial genomes downloaded from RefSeq) using the aligner BWA. The positive and unique aspect of this pipeline is that it considers the coverage of the bacterial genome using Bedtools and evaluates the expected vs. the observed coverage. By knowing how long the reads are and how much they should cover, DRAC computes an internal score to discard those reads with low coverage. By doing so, it penalizes and hence considers bacterial assignments with very poor coverage.

**Table 1.** Characteristics of the five benchmarked tools

Characteristics	*GATK PathSeq*	*Kraken2*	*MetaPhlAn2*	*DRAC*	*Pandora*
Status	Tool	Tool	Tool	Pipeline	Tool
Type	*Binner*	*Binner*	*Classifier*	*Binner*	*Classifier*
Input format	BAM	FASTQ	FASTQ	BAM	FASTQ
Aligner	BWA-MEM	—	BowTie2	BWA-ALN	—
Algorithm	Three subtractive filters	*K-*mer exact match	Marker genes	Coverage score	Assembly
Database size	25 917	12 409	7678	2546	—
Taxa	VBAF	VBA^a^	VBAF	B	VBAF

*Notes*: Quantities in ‘Database size’ row corresponds to the present species number. ‘Taxa’ row corresponds to the type of taxa recognizable by each tool.

V, viruses; B, bacteria; A, archaea; F, fungi.

a Kraken’s database was tailored to viruses, bacteria and archaea.

Pandora is a classifier tool based on multiple sequence alignment. Pandora takes as input the total RNA sequence reads from a sample and outputs detected microbial transcripts. The workflow is comprised of three major steps. First, the reads are mapped to the human host genome using both STAR and then bowtie2. Next, the remaining host-subtracted, unmapped reads are assigned a most likely species of origin using BLAST sequence searching in the nucleotide collection. Finally, the results from BLAST are filtered and converted into an interactive report.

### 2.2 Synthetic database construction

**Fig. 1.**
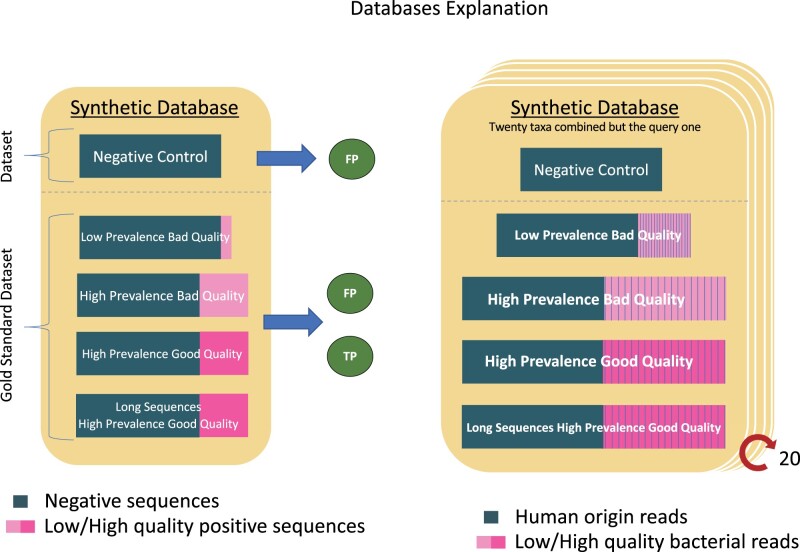
Example of the synthetic database structure. Database was formed of two datasets. The first dataset, (negative controls, on top) only with human sequences (represented by dark green) that will generate false positives exclusively. And the ‘Gold Standard’ dataset-2 (bottom) are composed both of human origin reads and bacterial reads (in pink). The Gold Standard dataset-2 is composed likewise by four files LPBQ, HPBQ, HPGQ and LSHPGQ files, indicated in the boxes. Right panel. Simplified structure of the database used for the benchmarking, in which the stripes represent the 20 species whose sequences are included in the simulated fastq files

The database was used as the ground truth to assess the tool’s performances. Sun *et al.* (2021) already stated that previous benchmarking studies have interchanged the two types of relative abundance (i.e. sequence and taxonomic) leading to unfair performance assessments. Therefore, in our study, we devise a subtraction strategy based on the two datasets of the synthetic database to use a unique metric for measuring the performance of all tools (i.e. binners and classifiers). To this end, we considered both possible sources of FP (i.e. bacterial and human origin). Figure 1 describes the database structure. The in-house MIME pipeline was used to this end. MIME is a python pipeline to simulate multiple microbial sequences like data files, it is available in https://github.com/fjuradorueda/MIME.

Sequence selection. The species selection process was a critical step as certain species sequences could either benefit or penalize the tool’s final performance. To avoid any bias, we selected a standard, common, and replicable set of species from the human microbiota. The 21 species were selected from the top abundance list identified by the Human Microbiome Project (Kraal *et al.*, 2014). We also annotated each species by interrogating the NCBI for gram category, phyla, absolute GC content, percentage of GC content, and genome length information. Human reads, included as negative control, came from initially unmapped TCGA RNA-Seq reads as our main interest was to compare the tools in a host RNA-Seq tissue setting. A read may be unmapped when the STAR, the aligner used by TCGA pipeline, did not map it to the human genome. We realigned these reads against the human genome again by using BWA thus ensuring that they were true human origin reads. The underlying idea of this procedure was to obtain conflicting reads (i.e. human origin but dismissed by STAR aligner) that could serve to test the performance of the tools when dealing with inconclusive sequences.

Conditions designed. The proportion of microbial vs. human reads was considered. We set up the proportion of bacterial reads according to two scenarios: ‘high-prevalence’ to be 10-fold greater than the ‘low-prevalence’ scenario with 10 000 and 1000 reads per microbe. Numbers were deliberately low to resemble a host RNA-seq dataset with few putative bacterial sequences. Specific-species prevalence was set to 3.9% and 1.5%; consequently, the overall bacterial prevalence was 78% and 30% to ‘high-prevalence’ and ‘low-prevalence’ scenarios, respectively. Regardless of the high or low proportion, both assays tried to simulate the low number of bacterial reads that might be present in a tumoral environment. All synthetic datasets were reproducing a paired-end sequencing assay. Regarding the base quality drop off, characters from the ASCII 33 list that encoded for quality were subtracted, and we also added a progressive penalization tail to the last 10 nucleotides of the sequence. These two steps were implemented in order to mimic the natural process of quality drop due to phasing and signal decay that usually affects the sequencing process (Anders and Huber, 2010). Mean quality was set up to 23 and 35 in the Phred scale for the ‘low-quality’ and ‘good-quality’ scenarios, meaning an average probability of error of 0.005 and 0.00032, respectively. Since quality value was derived from a probability distribution, there was a variance frame of size 4 (i.e. nucleotide quality could range until position 4 up or down from the mean in the Phred scale). As for the sequence-length, we selected two lengths: 48 (Short Sequence scenario, deliberately chosen to resemble as much as possible the TCGA RNA-Seq datasets) and 100 nucleotides for the Longer Sequences scenario. The genome sequence selected was another parameter that may influence the alignment-based algorithms, such as MetaPhlAn2, DRAC and GATK PathSeq. If the frame selected happened to be from a problematic region because of low-complexity or repetitive region, alignment-based tools would misclassify the reads. To solve this issue, we relied on a stochastic process of sequence selection. Our pipeline randomly selects a starting position, imitates the un-sequenced gap between the paired reads, and sets up a starting position for the reverse mate. The python script designed for the construction of these datasets is available in Supplementary Materials and in GitHub repository (Source code and detailed manual are available at https://github.com/fjuradorueda/MIME/).

### 2.3 Performance estimators

To measure the algorithm performances according to how well they classify the bacteria sequences, we calculated the true positive (TP), false positive (FP) and false negative (FN) rates against the known conditions in the synthetic database. These values were used to estimate each tool sensitivity (Se=TP/(TP+FN)), positive predictive value or precision (PPV=TP/(TP+FP)), and the F1 Score (F1 = 2*(Se*PPV)/(Se+PPV)) to measure accuracy. To discriminate TP from FP, we built two separate sets of files: Dataset (1), composed only of host (human) sequences and Dataset (2), a mixture of bacterial and human reads sequences, mimicking a real-world sample. Results obtained from running the tool on the Dataset-2 include both TP and FP, whereas those coming from the negative control Dataset-1 yields only FP. Subsequently, TP was calculated by subtracting Dataset-1 from Dataset-2 results. The synthetic Dataset-2 was built of four scenarios with changing conditions (i.e. microbiome prevalence, base quality and sequence length) (Fig. 1). The first scenario [Low Proportion and Bad Quality (LPBQ)] represented the worst conditions with low bacterial prevalence and bad base quality. The second scenario [High Proportion and Bad Quality (HPBQ)] simulated an intermediate state in which microbial prevalence was high but base quality was still bad. The third one, [High Proportion and Good Quality (HPGQ)], had high microbial prevalence and good base quality. Finally, the last one [Longer Sequences, High Proportion, and Good Quality (LSHPGQ)] gathered the best conditions of longer sequences, high microbial prevalence and a good base quality. In the synthetic Dataset-2, we launched 20 species mixed in the same file (i.e. all species except one) in each round, as shown in the right panel of Figure 1. The idea behind this procedure was to account for possible mishits across similar species. We annotated as FP any count given to the evaluated species (i.e. the missing species). In parallel, the same process was done using Dataset-1, composed of human origin sequences. Combining both approaches, we could identify the false reads coming from either human or bacterial origin. We named it as ‘exclusion database’ due to its strategy of leaving one species out in each instance. We scaled the process of datasets generation and tools launch up to 20-times in order to capture the variance resulting in twenty measures for every bacterium. The 20-times sensitivity average was utilized in the figures ahead due to the elevated similarity (i.e. and low variance) within the 20-times replicas.

### 2.4 Computational performance assessment

In order to evaluate the running time, CPU percentage and RAM memory usage, we set up three TCGA BAM files coming from Bladder, Melanoma and Pancreatic Cancer Datasets. These were the biggest files within each cancer type, with which those parameters could be better measured. Unmapped reads by the TCGA pipeline composed the three files (reads tagged with the bitwise flag 4: ‘read unmapped’). The command (/usr/bin/time -v) was used in order to measure these three parameters.

### 2.5 Statistical methods

We verified whether scores followed a parametric distribution or not through the Shapiro–Wilk test for small samples (i.e. <30). For larger sample size, Lillieforts version of Kolmogorov test was used. For the non-parametric scores, pair-wise Wilcoxon test was applied; whereas for parametric scores, we used *t*-test and ANOVA. Spearman’s rank correlation method was implemented when looking for correlations between the tool’s performance. We considered 0.05 as the upper limit for statistical significance.

## 3 Results

Outputs obtained from this effort regarded to the four condition-changing scenarios. All of them were launched to the three public available tools plus the two new pipelines (DRAC and Pandora) with these processes scaled up 20- times to capture variations. Thus, we ended up with a high-dimensional result. Below, we focused on the comparison across tools, scenarios and species to cement our objective.

### 3.1 Results across tools

**Fig. 2.**
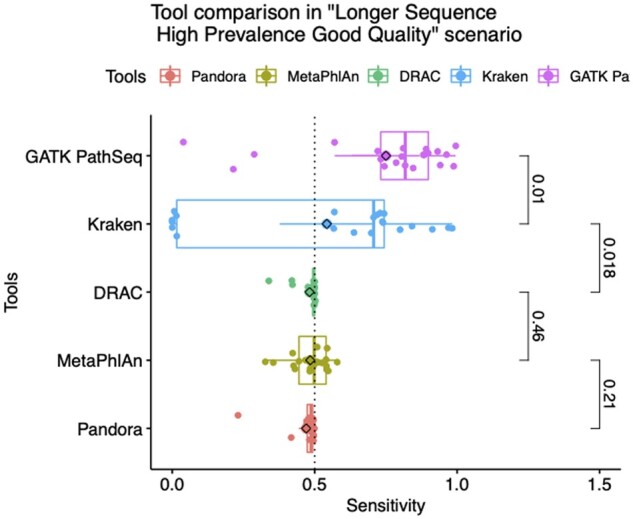
Sensitivity comparison of the five algorithms in the ‘Longer Sequences High Prevalence Good Quality’ trial. The dotted vertical line is set to 0.5. We also looked for differences in mean using non-parametric Wilcoxon rank-sum test, results were 0.01, 0.018, 0.46 and 0.21 respectively

This approach corresponds to the main objective of this paper, the tool performance benchmarking. Figure 2 shows results from the LSHPGQ scenario based on its real-world like conditions. By doing so, tools run in the best circumstances assuring a fair contrast. GATK PathSeq obtained the highest sensitivity (Se = 0.739, SD = 0.262), significantly different (*P*-value = 0.01) from Kraken2 with a Se = 0.543 (SD = 0.364) that itself was significantly (*P*-value = 0.018) greater from that of MetaPhlAn2. There were not significant differences of sensitivity among the last three algorithms. MetaPhlAn2 was ranked third (Se = 0.484, SD 0.065), followed by Pandora (Se = 0.470, SD = 0.057) and DRAC (SE = 0.459, SD = 0.038). On the other hand, DRAC showed the narrowest variance range of sensitivity (*P*-value = 0.038), followed by MetaPhlAn2 and GATK PathSeq, while Kraken2 showed the widest variance range, significantly different from DRAC and MetaPhlAn2. Considering the runtime, the computational speed ranking was headed by Kraken2 (runtime = 179 s, CPU = 9 percentage), followed by MetaPhlAn2 (runtime = 1,237 s, CPU = 82percentage). The slowest algorithm measured was Pandora (runtime = 131 793 s, CPU = 36.5percentage) (Supplementary Table S1).

### 3.2 Results across scenarios

**Fig. 3.**
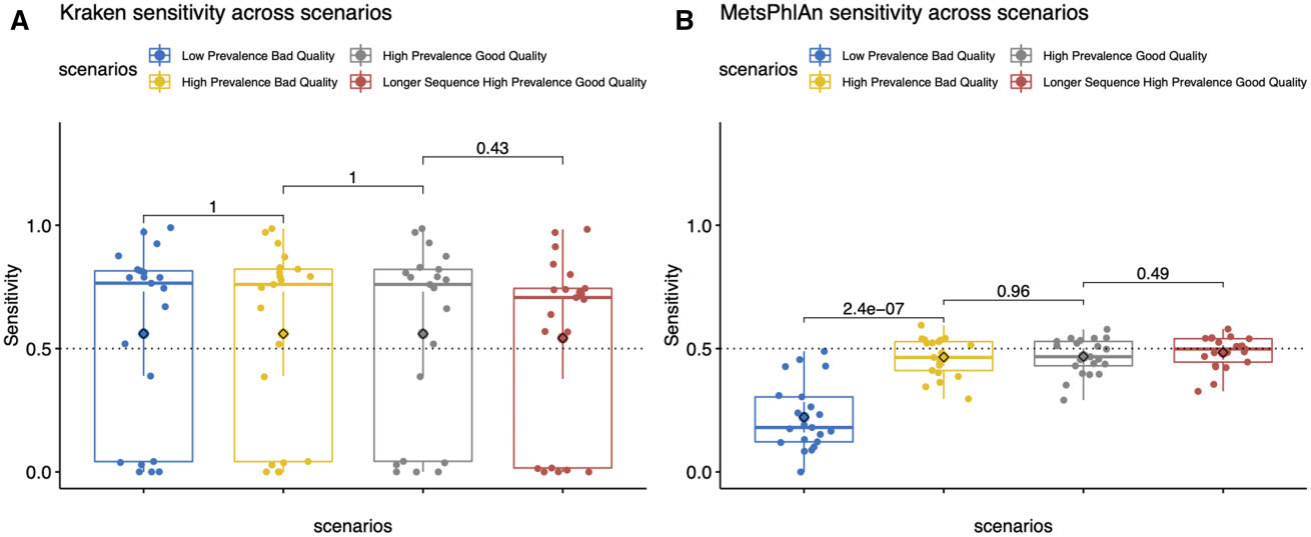
Kraken2 (left side) and MetaPhlAn2 (right side) sensitivity values a cross the four changing conditions scenarios. In the plot, median is represented by the line whereas mean is represented by the diamond. Wilcoxon rank-sum test was applied across scenarios

In this section, we describe how variation of sequence conditions did impact on tool’s performance. Graphs from this section gathered results from the 4 scenarios and for each tool. Scenarios were sorted condition-wise from left to right, meaning that prevalence, base quality, and sequence length were improved horizontally rightwards. Kraken2 demonstrated a strong robustness regardless the conditions variation, as shown in Figure 3A: Kraken2’s sensitivity did not improve when conditions were tuned, neither with read prevalence nor with nucleotide quality and read length. On the other hand, MetaPhlAn2 proved to be sensitive to bacterial sequence prevalence. As shown in Figure 3B, sensitivity mean was significantly different when the microbial prevalence increased in the second scenario (*P*-value = 2.4E−07). Bacterial proportion was the only condition to which MetaPhlAn2 improved its sensitivity. GATK PathSeq’s performance was comparable to Kraken2’s as its performance was quite robust across the four changing condition scenarios (Supplementary Fig. S1). No variation in the sequence’s nature seemed to modify its sensitivity. DRAC demonstrated to be the most sensitive tool to the changing conditions with nucleotide quality and sequence length significantly improving its sensitivity by 0.156 (*P*-value = 3.8E−08) and 0.164 (*P*-value = 3.8E−08), respectively (Supplementary Fig. S2). We observed that the narrow DRAC’s sensitivity distribution, previously seen in Figure 2, persisted in the rest of the three condition- changing scenarios. Pandora showed to be sensitive to read abundance. There was no variation in Pandora’s performance when improving the base call quality, nor the sequence length. Pandora’s algorithm performed significantly good when classifying reads of bad quality present in a low proportion (see Supplementary Figure S3).

### 3.3 Results across species

**Fig. 4.**
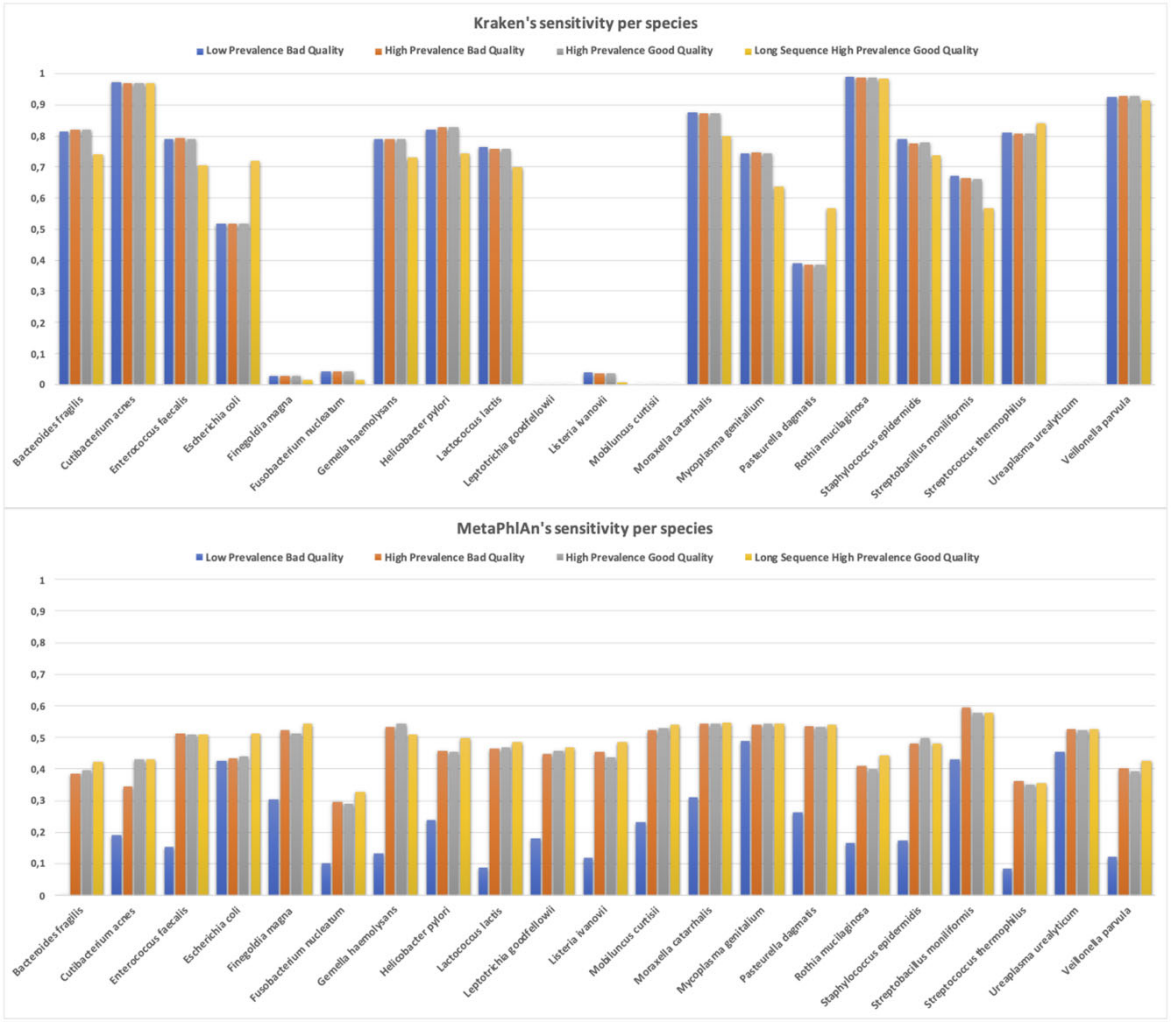
Species’ sensitivity values within each of the four scenarios. Kraken2 (top) and MetaPhlAn2 results (bottom). The four scenarios considered were sorted from left to right condition-wise. Species were sorted alphabetically

The classification ability clearly varied throughout species. Kraken2’s ability to classify each species did not vary generally across the four scenarios. The most relevant aspect of Figure 4A is the sensitivity boost of *Escherichia coli* and *Pasteurella dagmatis* when the length of the reads was elongated (*P*-values <2.22E−16). Supplementary Figure S4 shows how significant was the increase in number of counts that these two species suffered. MetaPhlAn2 results showed a wide variation of sensitivity depending on the species. In Figure 4B, we can see that for all 21 species, sensitivity was greater in the high-prevalence scenarios. However, certain species performance did not react to further changes according to base quality and sequence length. Notice that the low sensitivity seen in Figure 3B in the LPBQ scenario persisted across all species. This means that all species suffered from the same sensitivity shortage when the prevalence was low. Regarding DRAC’s output, all species showed a very similar sensitivity patterns according to the different condition-changing scenarios. All of them improved their sensitivity when increasing the sequencing quality and read length. None of the 21 species showed a different behavior as displayed in Supplementary Figure S5. As of GATK PathSeq, we noticed that species clustered by sensitivity within three clear groups when sorting by any of the three first scenario’s sensitivity (Supplementary Figure S6B). In Supplementary Figure S6A we observe that results for E. coli significantly differed between the last and the three first scenarios (*P*-value = 1.50E−23). The same phenomenon was observed among Kraken2’s results (see above). Pandora’s results per species were displayed in Supplementary Figure S7. No clear differences among species were observed, besides the slight sensitivity rise of *E.coli* and *P.dagmatis* when the reads were elongated.

### 3.4 Tool’s performance correlation

Pandora and Kraken2 sensitivity showed significant positive correlation (Rho = 0.540, *P*-value = 0.012), as these two tools agreed moderately on the species detection (Table 2). It is worth mentioning the notable negative correlation between MetaPhlAn2 and Kraken2 sensitivity (Rho = −0.373, *P*-value = 0.096) meaning that both tools identified species at a different rate from the same sample file.

**Table 2.** Spearman correlation between tools based on sensitivity to detect species from the LSHPGQ scenario

Algorithms	*Kraken2*	*MetaPhlAn2*	*GATKPathSeq*	*DRAC*	*Pandora*
*Kraken2*	—	0.096	0.889	0.433	0.012
*MetaPhlAn2*	−0.373	—	0.089	0.048	0.389
*GATK PathSeq*	0.033	0.381	—	0.086	0.120
*DRAC*	−0.180	0.435	0.383	—	0.741
*Pandora*	0.540	−0.198	0.350	−0.08	—

*Notes*: Salmon background cells contain Rho values, whereas blue background cells contain the correspondent *P*-values.

## 4 Discussion

In this study, we benchmarked the performance of five algorithms for bacterial sequence identification across multiple conditions according to four synthetic scenarios in which tools had to deal with three remodeling conditions according to base quality, species prevalence and sequence length. To the best of our knowledge, only CAMISIM (Fritz *et al.*, 2019) and MSC (Saha *et al.*, 2019) performed similar analyses. The former paper tuned the error rate but only on two assembly tools. In the second paper, two scenarios of good Phred quality score (30) and bad Phred quality score (10) were considered, improving some of the tested tools’ performance (i.e. same pattern found among our results). However, this finding was not statistically tested, nor mentioned in the paper. In addition, our tool’s ranking regarding RAM percentage and time required, fully agreed with preceding publications (Ye *et al.*, 2019). Concerning the runtimes, Kraken2 has proven to be one of the fastest in this comparison. This result agrees with those from other reports that assessed sequence classifiers, among them Pathoscope (Andrusch *et al.*, 2018). The a priori information of how the tool performs upon the data characteristics is key for researchers to know which tool/s should be applied and how to interpret results. GATK PathSeq showed to be the best tool in terms of sensitivity, it also performed robustly across the scenarios. However, the main drawback of this tool was its slowness. Kraken2 was the second-best tool in terms of sensitivity. Nevertheless, its main limitation was the wide sensitivity variance as its performance depended on the species to be identified. The fact that Kraken2 negatively correlated with MetaPhlAn2, may make them complementary strategies. The fact that MetaPhlAn2 maps the fewest reads due to the methodology based on sets of marker genes, while Kraken2 maps >70% have been previously reported (Lindgreen *et al.*, 2016). MetaPhlan2 was ranked third regarding sensitivity; nonetheless, this tool showed some limitations when dealing with low species prevalence samples, as Pandora does, too. The fact that the other tools are not affected by the species prevalence may be because binner tools work on a read-by-read basis and the number of reads does not really affect the sensitivity. Regarding Kraken2, it is based on k-mer exact matches and, therefore, the tool disregards the total read’s prevalence. The strength of DRAC was its narrow variance across species sensitivity, as all species were equally recognized. DRAC’s sensitivity was barely as good as MetaPhlAn2 and Pandora. The main limitations of DRAC regarded to the low base call quality and the short reads conditions. Finally, the main benefit of Pandora over the other tools lies in superior performance under low-quality data conditions. We found no significant associations between any species sensitivity and their genome features for the tested tools, as shown in Supplementary Table S2. This suggests that the pattern of results displayed in Supplementary Figure S6 is possibly an artifact as we cannot explain the results shown for *E.coli* and *P.dagmatis* in the best scenario for Kraken2 (Supplementary Fig. S4), GATK PathSeq and DRAC. Several methodological aspects need to be considered in interpreting the results of this study. In building the synthetic database, we reproduced the real world-like 1percentage (average) of unmapped reads resembling TCGA RNA-Seq data; though we knew that this was a parameter that would differentially affect the tool’s activity. DRAC, for instance, initially discarded any human-mapped read, however GATK PathSeq tried to remap all the reads. Binner tools disregarded the total amount of sequences of a file; instead, they iterated one by one applying their algorithms to classify the microbe, focusing on one at a time. This fact means that the proportion of the microbe reads was rather tentative and guided binners’ results. We are aware that species selection was a decisive step for obtaining a trustable analysis and further comparable results among tools. In building the synthetic database, all selected species were represented. We observed the phenomenon of k-mer-based tools struggling with the identification of extensively studied model organisms. Since Kraken2 deals with smaller units (k-mers) of the queried sequence, it is hard for the algorithm to distinguish between so many genomes available for the same species (e.g. *E.coli*, which showed a noticeable decrease in the sensitivity). As a result, Kraken2 frequently labels the k-mer and, subsequently, the read to the LCA (Nasko *et al.*, 2018). When host tissue or tumor RNA-Seq data is available, we propose to apply tools prioritized upon its sensitivity in a first filtering step in microbe identification, previous to their validation with more specific approaches (i.e. 16S rRNA sequencing). PPV was not suitable for tool ranking since it was at its top values in all tools due to the low FP rates. In general, detection tools yielded almost no FP, meaning high precision values. In contrast, FN rates were larger and more varied, a fact that was translated into a lower and more dispersed sensitivity rates. Ultimately, we focused on sensitivity alone for the sake of simplicity, since precision and F1 score provided no further value or interpretation (Supplementary Fig. S8). In this study, we sequentially considered three factors (i.e. reads prevalence, base call quality and sequence length) potentially affecting the host tissue or tumor microbe identification using RNA-Seq data: namely, we tested five tools against a database of 21 bacterial species, under four different conditions/scenarios, with 20 replicates each resulting in a tool ranking in terms of sensitivity. We also considered information regarding how the tools performed and dealt with changing-conditions scenarios. However, other factors potentially affecting results, such as mutations, strains, other contaminants could not be considered due to the technical constraints in building databases. Nevertheless, these are key issues to be considered in future benchmarking papers. In summary, GATK PathSeq was the best tool in terms of overall sensitivity, Kraken2 ranked the second, and MetaPhlan2, DRAC and Pandora performed similarly in a third position. However, based on the balance between low runtime and competitive sensitivity, we would recommend Kraken2 to be applied first in microbe identification using RNA-Seq data. Nevertheless, we strongly endorse the use of two tools in tandem according to the data conditions mentioned above. Results could then be selected through a more sensitive approach if all species identified by the two tools are considered (i.e. Kraken2 plus MetaPhlAn2) due to their complementarity in the species identification, or through a more specific strategy if results are restricted to only those identified species by the two tools (i.e. GATK PathSeq and DRAC).

## Supplementary Material

vbad014_Supplementary_Data
