# Correction to: Benchmarking of microbiome detection tools on RNA-seq synthetic databases according to diverse conditions

**DOI:** 10.1093/bioadv/vbad183

**Published:** 2023-12-26

This is a correction to: Francisco Jurado-Rueda, Lola Alonso-Guirado, Tomin E Perea-Chamblee, Oliver T Elliott, Ioan Filip, Raúl Rabadán, Núria Malats, Benchmarking of microbiome detection tools on RNA-seq synthetic databases according to diverse conditions, *Bioinformatics Advances*, Volume 3, Issue 1, 2023, vbad014, https://doi.org/10.1093/bioadv/vbad014

In the originally published version of this manuscript, one of the authors’ surnames was misspelled. The correct surname for the third author is “Perea-Chamblee” and not “Perea-Cham-Blee”.

This error has been corrected online.

