# Supplementary material for: Benchmarking of microbiome detection tools on RNA-seq synthetic databases according to diverse conditions
Source: Bioinform Adv. 2023 Feb 22;3(1):vbad014. doi: 10.1093/bioadv/vbad014 (PMC9976984; doi:10.1093/bioadv/vbad014)

Supplementary Materials
Jurado-Rueda F, *et al*. Benchmarking of microbiome detection tools on RNA-Seq synthetic databases with diverse conditions

Supplementary File I – Tools commands and software versions

Supplementary File II - *DRAC* (Discarded Reads Alignment and Coverage) pipeline

Supplementary Table S1: Spearman correlation between species' sensitivity and its correspondent length and GC content. Values correspond to the Rho estimation.
Supplementary Table S2: Running specificities: time in seconds, CPU percentage, and Maximum resident set size (RAM) across the 5 tools. The second column refers to the number of human genome unmapped reads

Supplementary Figure S1: GATK PathSeq sensitivity values a cross the 4-changing conditions scenarios. In the plot, the median is represented by the line whereas the mean is represented by the diamond. Wilcoxon rank-sum test was applied across scenarios.

Supplementary Figure S2: *DRAC* sensitivity values across the 4-changing conditions scenarios. In the plot, the median is represented by the line whereas the mean is represented by the diamond. Wilcoxon rank-sum test was applied across scenarios.

Supplementary Figure S3: *PANDORA* sensitivity values a cross the 4-changing conditions scenarios. In the plot, the median is represented by the line whereas the mean is represented by the diamond. Wilcoxon rank-sum test was applied across scenarios

Supplementary Figure S4: T-test applied to kraken's results. The "Low prevalence Bad Quality" scenario was not included to the comparison because it cannot be compared with the rest of the high prevalence scenarios (i.e., sequence's number is tenfold smaller).

Supplementary Figure S5: Species sensitivity values within each of the 4-changing conditions scenarios using *DRAC*. The four scenarios considered are sorted from left to right condition wise. Species were sorted alphabetically.

Supplementary Figure S6: Species sensitivity values within each of the 4-changing conditions scenarios using *GATK PathSeq*. The four scenarios considered are sorted from left to right condition wise. (A) Species were sorted alphabetically. (B) Species were sorted according to the first scenario's sensitivity.

Supplementary Figure S7: Species sensitivity values within each of the 4-changing conditions scenarios using *Pandora*. The four scenarios considered are sorted from left to right condition wise. Species were sorted alphabetically.

Supplementary File I: Tools commands and software versions

All programs were executed in 8-threads mode in a workstation with 32 Intel(R) Xeon(R) CPU, with 96 GB of RAM memory, running Ubuntu 20.04.4 LTS (Focal Fossa)

Run Kraken: kraken version 2.1.2

```
kraken2 --threads 8 --db /local/databases/refSeqBacArchVir/refSeqBAVkraken --output readbasis_ow.txt --report-zero-counts --paired --confidence 0.90 --report krakenout.tmp ./fg_1.fq ./fg_2.fq

sort -k5,5n krakenout.tmp | awk 'BEGIN{FS="\t"; OFS="\t"} {if ($4 == "S") print $5,$2,$3,$6 }' | sed 's/\t */\t/g' > krakenout.krk
```

Run MetaPhlAn: MetaPhlAn version 2.6.0

```
metaphlan2.py fg_1.fq,fg_2.fq --bowtie2out gs.bowtie2.bz2 -t rel_ab_w_read_stats --ignore_viruses --ignore_archaea --nproc 8 --input_type multifastq > profiled_gs.txt
```

Run PathSeq: gatk-4.2.0.0

```
nombre=gold_sd_prb
/local/fjurado/gatk-4.1.2.0/gatk PathSeqPipelineSpark --input out1.bam --min-base-quality 1 --min-score-identity 0.5 --identity-margin 0.02 --filter-bwa-image /local/fjurado/pathseq_ref_files/pathseq_host.fa.img --kmer-file /local/fjurado/pathseq_ref_files/pathseq_host.bfi --min-clipped-read-length 48 --disable-tool-default-read-filters true --microbe-fasta /local/fjurado/pathseq_ref_files/pathseq_microbe.fa --microbe-bwa-image /local/fjurado/pathseq_ref_files/pathseq_microbe.fa.img --taxonomy-file /local/fjurado/pathseq_ref_files/pathseq_taxonomy.db --output $nombre.bam --scores-output $nombre.txt 2>$nombre.log
```

Supplementary File II: *DRAC* (Discarded Reads Alignment and Coverage) pipeline

DRAC is a pipeline that takes a sequencing file as input and scans for bacterial sequences in it. *DRAC* shares the same conceptual framework than other similar pipelines: RNA CoMPASS (Xu et al 2014), PHAT (Pathogen-Host Analysis Tool, Gibb et al. 2019), PAIPline (Andrusch et al., 2018) and SLIMM (Dadi et al., 2017) among others. The dual study of pathogen and host genome/transcriptome from NGS files is an application that has gained interest in the scientific community, because two feature layers can be extracted in parallel. For instance, from a unique RNA-Seq file one can study the host transcriptome and the discovery of pathogens as well (see Xu et al 2014). Pathogen-host NGS analysis allows detection of microbes without prior hypothesis about possible causative/contaminant agents.

The workflow *DRAC* follows involves several consecutive steps: sequence alignment to the host genome, read cleaning and filtering, mapping against a database of interest (RefSeq representative genomes), and on top of that, *DRAC* computes the coverage along every reference genome. That said, *DRAC* is not a metagenomics classifier but, instead, a pipeline that was developed to scan NGS files for putatively present microbiota or contaminant microbes.

DRAC is conceived as a post-processing tool to be run on a NGS file (either fastq or SAM/BAM format) of previously discarded reads that could not be aligned to the host genome (it may be human or any other host). It is mostly devised to be run on a single organism tissue RNA-Seq, but also Whole Exome Sequencing or Whole Genome Sequencing are supported, rather than a high-biomass metagenomic design (i.e., it is not recommended for high biomass studies, e.g., feces or saliva). This is because the program that *DRAC* uses for identifying the microbes is the Burrows-Wheeler Aligner (BWA) that it is not designed for that sort of task, besides of being slow due to the alignment process. However, BWA its very appropriate to align a relatively small subset of Illumina single/paired-end sequences from previously aligned reads to the human/host reference genome, against the complete bacterial genomes in the RefSeq database, as was performed in a seminal paper that identified bacteria that may be present in human tumor samples (Robinson et al 2017, DOI: 10.1186/s40168-016-0224-8)

Since with BWA identifications one cannot conclude whether the reads are true or false positives, DRAC pipeline subsequently applies a method to ensure that counts belonging to a particular genome are well spread and covering a directly proportional part of it, as a proof that they are more reliable.

DRAC is a binner tool that takes a BAM file as input (with a minor change it can start from a non-host reads fastq file) keeping both the host aligned reads and the unmapped (discarded by the aligner, for whatever reason) that DRAC classifies by the bitwise flag (samtools). Afterwards, DRAC trims the still remaining adapters, the low complexity sequences with the DUST algorithm, and the low quality tails, and dismisses the short reads after this process, saving the clean ones in a fastq file.

Since the remaining reads are unsorted and one of the pairs members is sometimes lost, the pipeline fixes the pairs to keep only the paired reads. It also saves the singletons in case the user needs to analyze them in a further step.

BWA aligns the unmapped but sorted paired-end reads against a reference database. In the article that we present here, a database was previously built (BWA-indexed) with the bacterial genomes downloaded from RefSeq and, in particular, the set of Representative Genomes (one assembly per defined species selected as representative, with criteria explained in https://www.ncbi.nlm.nih.gov/refseq/about/prokaryotes/#representative_genomes). Other similar pipelines (e.g., SLIMM performs a species genome selection when multiple genomes are available).

After aligning the reads, the program extracts the counts per genome and joins them in a sample counts file. The pipeline can also join the counts of several samples in one single table. Afterwards, *DRAC* uses bedtools coverageBed for computing the coverage along the genomes represented in the sequence alignments of the resulting BAM file. The computed coverages are

joined in a sample file (again, individual sample or merged when there is a group of samples in the analysis).

A unique aspect of *DRAC* pipeline is that it takes into account the coverage of a particular bacterial genome and evaluates the expected vs. the observed coverage. DRAC assumes a constant read length (the average length, also calculated by the method), and it also assumes that reads should be widespread or scattered along the genome for them to be considered reliable, similar to KrakenUniq and SLIMM, discarding unlikely genomes based on coverage landscape. This way, those methods eliminate genomes that have a stack of reads only in few places across their genomes, which could be a result of sequencing artifacts, spike-ins, or a conserved region among distant relatives.

By knowing how long the reads are and how much they should cover if they are fairly spread (Expected Coverage = read length x number of reads), we can calculate an internal score to discard those reads with low coverage (according to what it would be expected by the abovementioned formula). By doing so, *DRAC* penalizes bacterial assignments with very poor coverage and dismisses them, while it will retain the most reliable (scattered through the genome) ones, that most probably are true positives.

The pipeline also performs some calculations, proportions, and creates a global table in case of scanning several samples. In the last stages, DRAC performs the count normalization and statistical analysis.

DRAC scripts are written in Bash, but it also has pieces of code in Python, and R languages, and the project can be found on the GitHub repository (https://github.com/lola4/DRAC), as well as documentation and explanatory diagrams (Supplementary Figures S9 and S10).

References cited in Annex 2 - *DRAC* (not included in the main text)

https://doi.org/10.1093/bioinformatics/bty1003
Pathogen–Host Analysis Tool (PHAT): an integrative platform to analyze next-generation sequencing data (Gibb et al 2018).

DOI: 10.1186/s40168-016-0224-8
Distinguishing potential bacteria-tumor associations from contamination in a secondary data analysis of public cancer genome sequence data (Robinson et al. 2017).

https://doi.org/10.1186/s13059-018-1568-0
KrakenUniq: confident and fast metagenomics classification using unique k-mer counts. (Breitwieser, et al 2018).

https://doi.org/10.1371/journal.pone.0089445
RNA CoMPASS: A Dual Approach for Pathogen and Host Transcriptome Analysis of RNA-Seq datasets (Xu et al 2014).

Supplementary Table S2: Spearman correlation between species' sensitivity and its correspondent length and GC content. Values correspond to the Rho estimation.

\	Genome Length	*p*-value	GC Content absolute	*p*-value
Kraken	0.220	0.380	0.209	0.537
MetaPhlAn2	-0.426	0.078	-0.245	0.467
GATK PathSeq	-0.191	0.448	-0.045	0.894
DRAC	-0.500	0.035	-0.278	0.408
Pandora	0.064	0.784	0.655	0.029

Supplementary Table S2: Running specificities: time in seconds, CPU percentage, and Maximum resident set size (RAM) across the 5 tools. The second column refers to the number of human genome unmapped reads.

TCGA file	Number of reads	*GATK PathSeq*	*Kraken*	*Metaphlan2*	*DRAC*	*Pandora*
Bladder	36,707,100	1,635s<br>22%<br>78.9 GB	179s<br>9%<br>44GB	1,237<br>82%<br>1.4GB	31,675s<br>94,9%<br>17.5GB	131,793s<br>36,5%<br>15GB
Pancreas	17,539,806	1,530s<br>21.5%<br>78.9 GB	76.2s<br>11.2%<br>44GB	633s<br>70%<br>1.3GB	13,116s<br>87%<br>17.16GB	115,200s<br>16,9%<br>15GB
Melanoma	24,799,394	2,216s<br>23%<br>79 GB	97.2s<br>9.8%<br>44GB	1003.3s<br>84.5%<br>1.3GB	9,291s<br>80%<br>17.5GB	111,600s<br>24.6%<br>15GB

Supplementary Figure S1: *GATK PathSeq* sensitivity values a cross the 4-changing conditions scenarios. In the plot, the median is represented by the line whereas the mean is represented by the diamond. Wilcoxon rank-sum test was applied across scenarios.

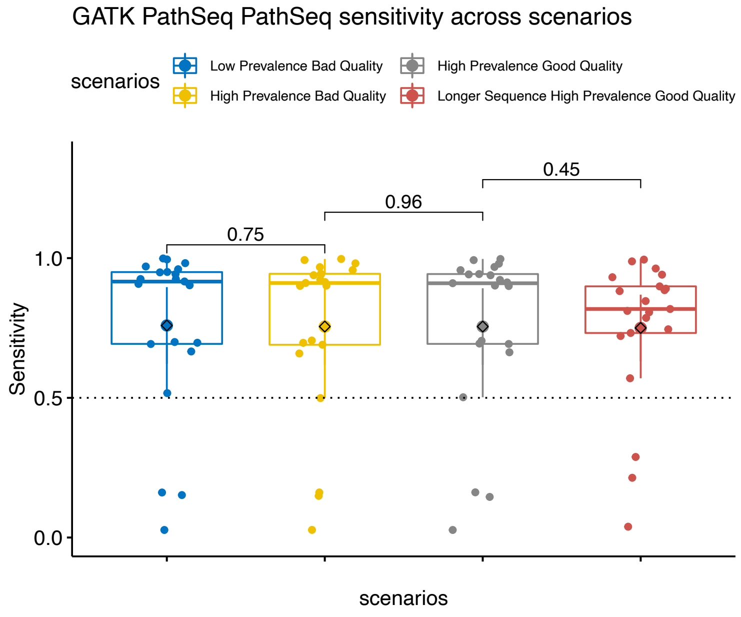

Supplementary Figure S2: *DRAC* sensitivity values across the 4-changing conditions scenarios. In the plot, the median is represented by the line whereas the mean is represented by the diamond. Wilcoxon rank-sum test was applied across scenarios.

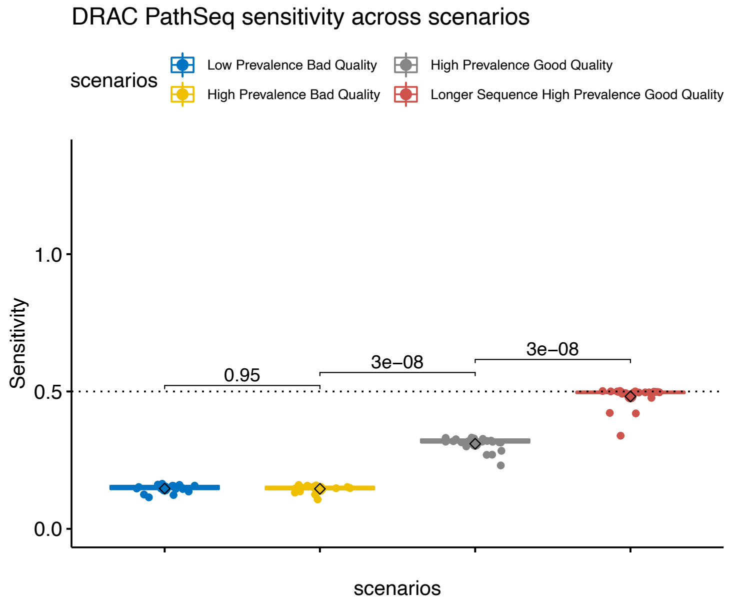

Supplementary Figure S3: *PANDORA* sensitivity values a cross the 4-changing conditions scenarios. In the plot, the median is represented by the line whereas the mean is represented by the diamond. Wilcoxon rank-sum test was applied across scenarios

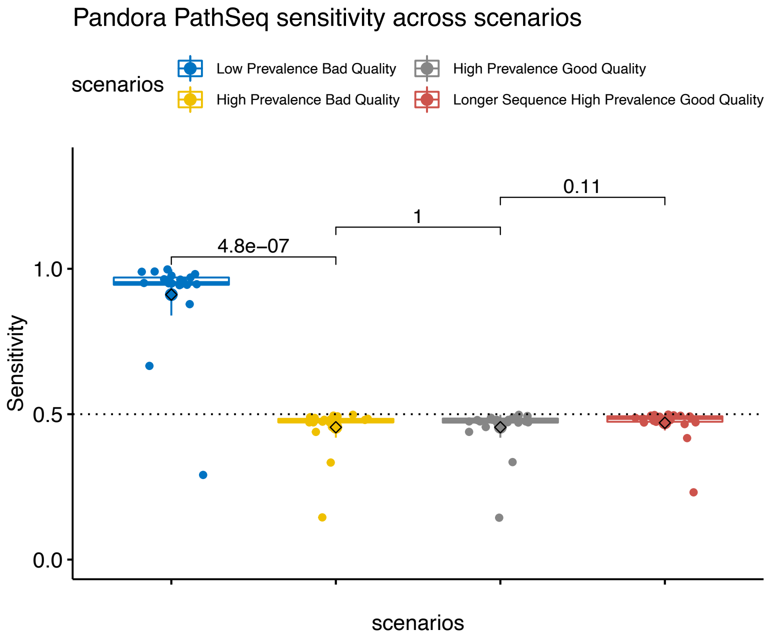

Supplementary Figure S4: T-test applied to kraken's results. The "Low prevalence Bad Quality" scenario was not included to the comparison because it cannot be compared with the rest of the high prevalence scenarios (i.e., sequence's number is tenfold smaller).

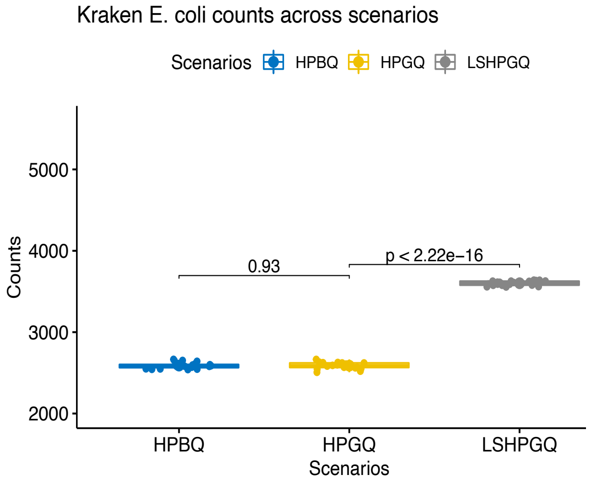

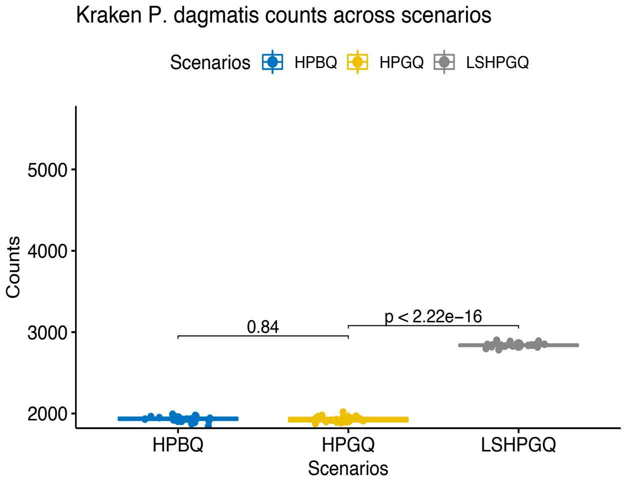

Supplementary Figure S5: Species sensitivity values within each of the 4-changing conditions scenarios using *DRAC*. The four scenarios considered are sorted from left to right condition wise. Species were sorted alphabetically.

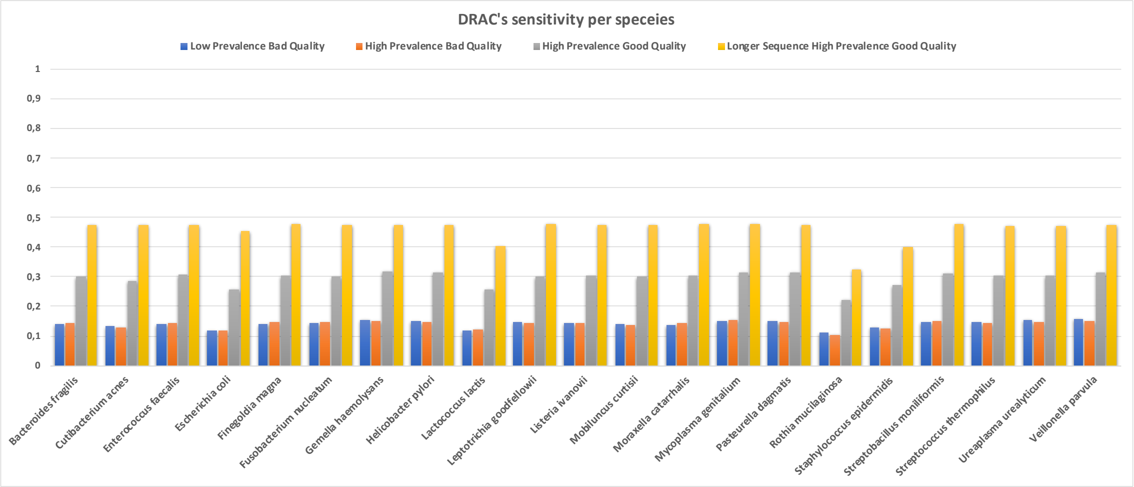

Supplementary Figure S6: Species sensitivity values within each of the 4-changing conditions scenarios using *GATK PathSeq*. The four scenarios considered are sorted from left to right condition wise. (A) Species were sorted alphabetically. (B) Species were sorted according to the first scenario's sensitivity.

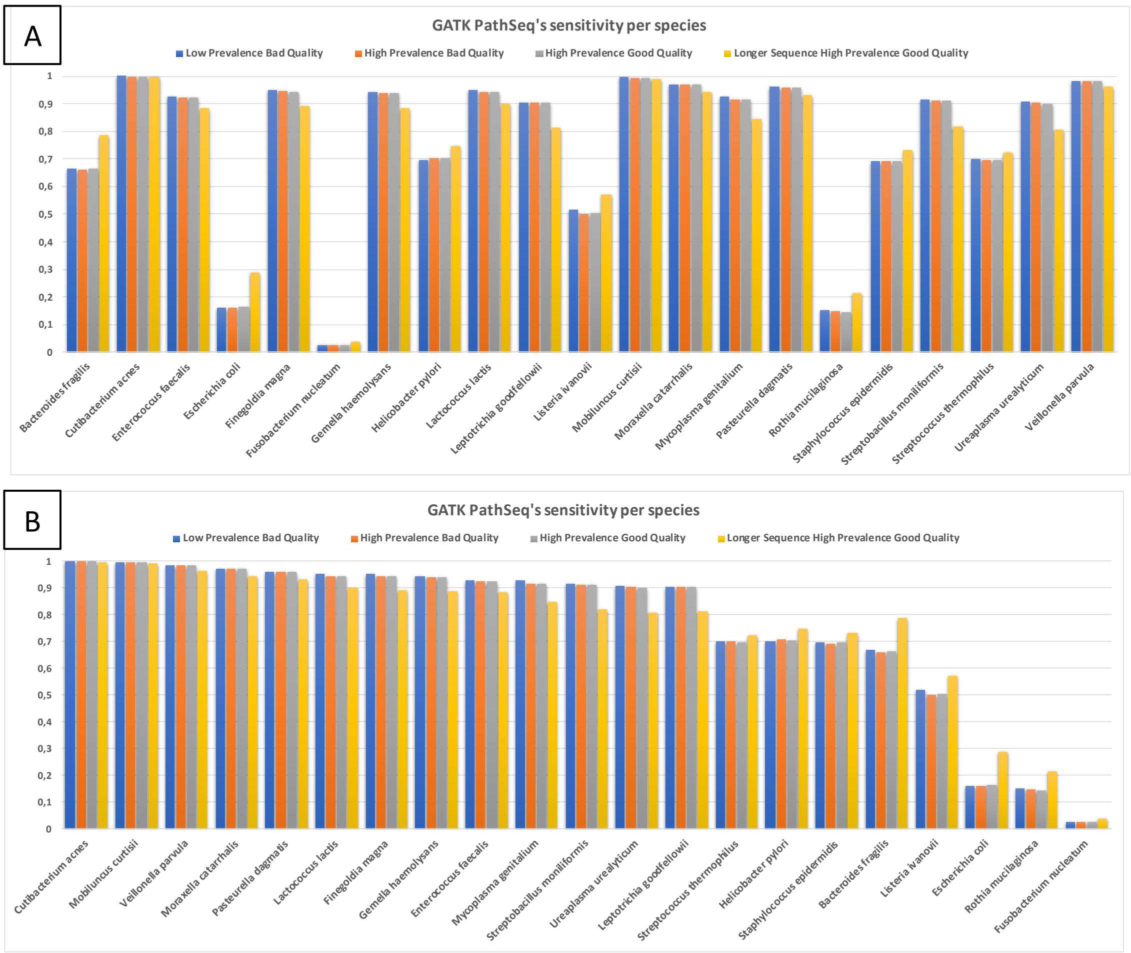

Supplementary Figure S7: Species sensitivity values within each of the 4-changing conditions scenarios using Pandora. The four scenarios considered are sorted from left to right condition wise. Species were sorted alphabetically.

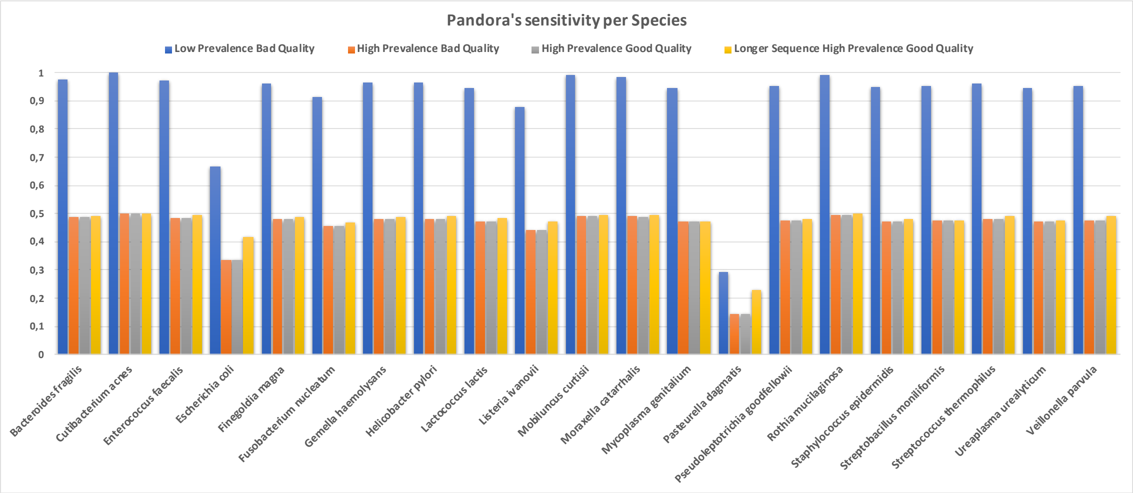

Supplementary Figure S8: F1 score and precision comparison of the 5 algorithms in the "Longer Sequences High Prevalence Good Quality" trial.

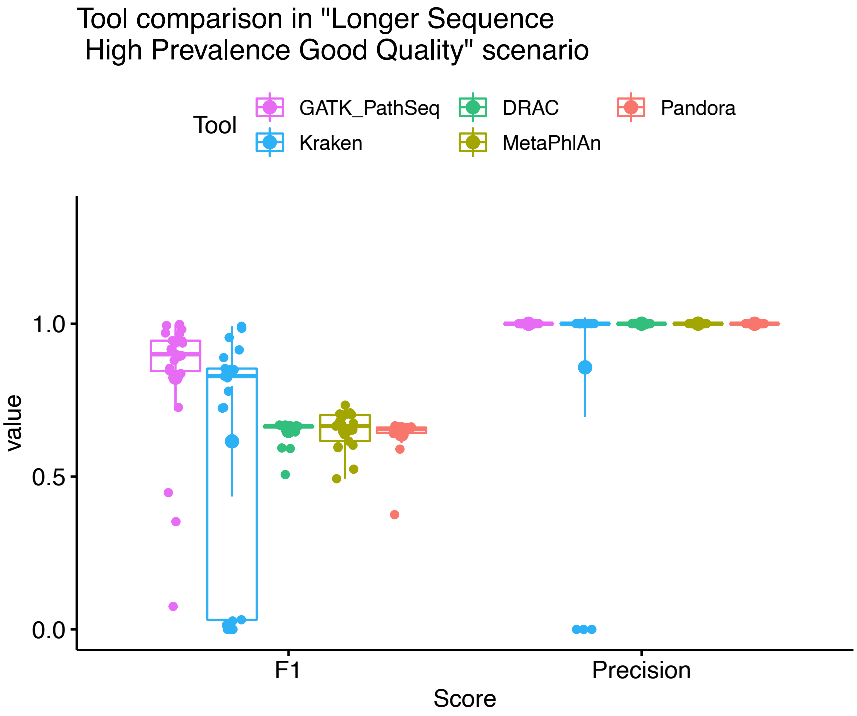

Supplementary Figure S9: Overview of the *DRAC* pipeline showing the consecutive stages of the workflow. Below, two examples of different coverage and reliability. *Bottom left:* a stack of reads aligning to the same genome region is shown (Integrative Genomics Viewer snapshot), along 6 different BAM files, which would be an example of poor coverage and not reliable microbe. *Bottom right:* IGV snapshot showing 5 BAM files with reads aligning to the same genome region, but in this case the reads are scattered and widespread, which is a proof of reliability following the assumptions of the method.

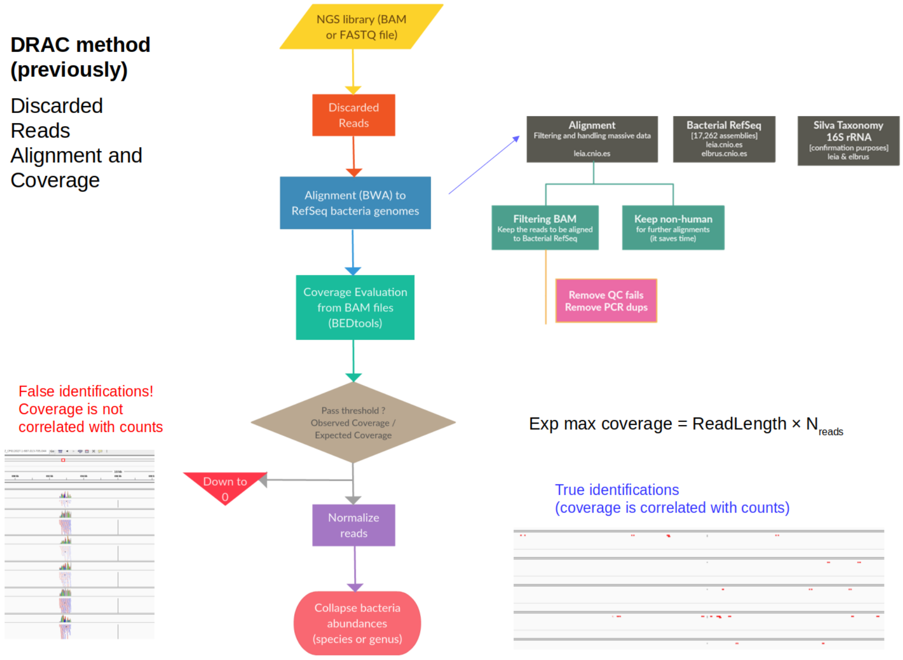

Supplementary Figure S10: *DRAC* penalization scheme showing an example of a counts table (*left:* samples in columns, bacteria in rows) and a coverages table calculated by bedtools coverageBed (*right:* samples in columns, bacteria in rows). The blue matrix shows the Expected coverage, which results from multiplying the counts table by a scalar, the average read length (e.g., 100 bp). The Observed / Expected table is computed by the element-wise division of two matrices, the Observed coverageBed table, by the Expected values. This new matrix elements have a range of 0 to 1, being values close to zero, genomes with poor coverage (non-trustworthy) and values close to one genome with high coverage (reliable microbes). The software needs a user-provided threshold to decide the genomes that will be penalized down to zero. In this example and for most applications, a value of 0.4 or higher, is recommended, being threshold values close to 1 the most restrictive ones, while on the contrary, a 0 value for the threshold will not penalize at all. This threshold will be used as the cutoff value to create a new binary matrix, with only 0 and 1 values, being the 0 elements, genomes that will be dismissed, and 1, genomes that will be trusted. The binary matrix will multiply the observed counts table to yield the final count table for downstream statistical analyses.

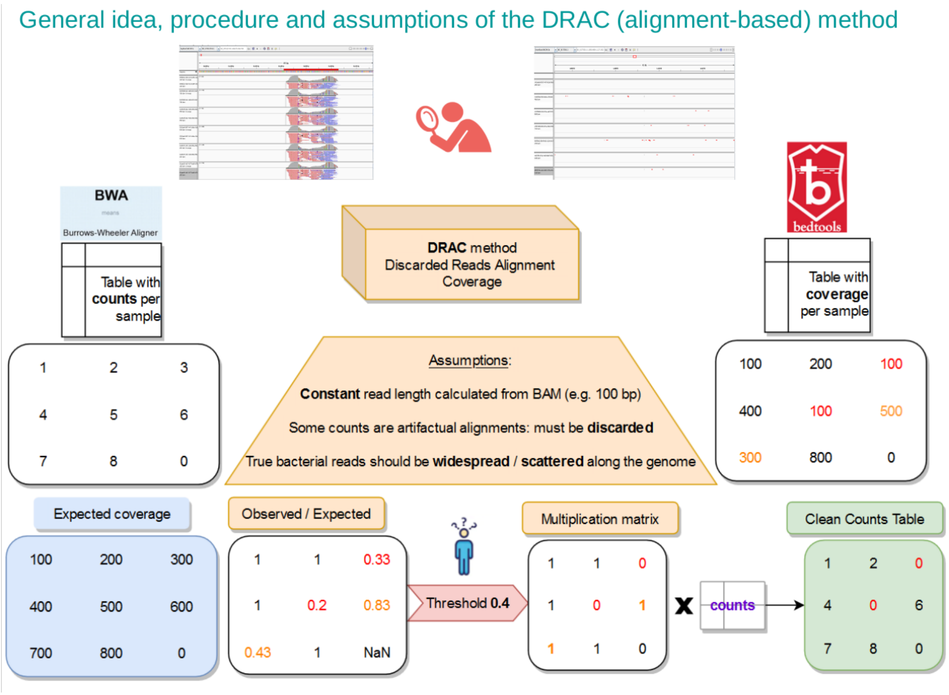

Supplement: vbad014_Supplementary_Data [file vbad014_supplementary_data.pdf]
